# Rosai–Dorfman disease mimicking IgG4-related diseases: a single-center experience in China

**DOI:** 10.1186/s13023-020-01567-6

**Published:** 2020-10-14

**Authors:** Li Wang, Wei Li, Shangzhu Zhang, Linyi Peng, Min Shen, Shuoning Song, Wei Zhang, Xinxin Cao, Ruie Feng, Wen Zhang

**Affiliations:** 1 Departments of Rheumatology and Clinical Immunology, Peking Union Medical College Hospital, Chinese Academy of Medical Sciences & Peking Union Medical College, The Ministry of Education Key Laboratory, National Clinical Research Center for Dermatologic and Immunologic Diseases, Beijing, 100730 China; 2 Department of Pulmonary and Critical Care Medicine, Center of Respiratory Medicine, China-Japan Friendship Hospital; National Center for Respiratory Medicine; Institute of Respiratory Medicine, Chinese Academy of Medical Sciences; National Clinical Research Center for Respiratory Diseases; WHO Collaborating Centre for Tobacco Cessation and Respiratory Diseases Prevention, Beijing, 100029 China; 3 Departments of Endocrinology, Peking Union Medical College Hospital, Chinese Academy of Medical Sciences & Peking Union Medical College, Beijing, 100730 China; 4 Departments of Haematology, Peking Union Medical College Hospital, Chinese Academy of Medical Sciences & Peking Union Medical College, Beijing, 100730 China; 5 Departments of Pathology, Peking Union Medical College Hospital, Chinese Academy of Medical Sciences & Peking Union Medical College, Beijing, 100730 China

**Keywords:** Immunoglobulin G4-related disease, Histiocytosis, Pathology, Rosai-Dorfman disease, Prognosis

## Abstract

**Background:**

Rosai–Dorfman disease (RDD) and IgG4-related (IgG4-RD) disease are both rare diseases, but in some cases, RDD mimics IgG4-RD clinically and pathologically. RDD mimicking IgG4-RDs (RDD mimic IgG4-RD), referring to disease initially diagnosed as IgG4-RD but finally pathologically confirmed to be RDD, is a clinically rare and confusing disease. To summarize the characteristics of this disease, we prospectively analyzed the clinical features, laboratory parameters, pathological characteristics, treatment and prognosis of patients diagnosed with RDD mimic IgG4-RD. Moreover, by analyzing characteristics of RDD mimic IgG4-RD, RDD and IgG4-RD, we further compared the similarities and differences between RDD and IgG4-RD.

**Results:**

7 patients with RDD mimic IgG4-RD were included in this study and all of them had extranodal organ involvement, especially the central nervous system, which occurred in 5 patients (71.4%). Although serum IgG4 level was elevated in 6 cases (1360–54,100 mg/L), overall, it was still lower than that in IgG4-RD patients. Furthermore, we found a new cut-off value of serum IgG4 concentration for differentiating RDD and IgG4-RD with higher specificity. Pathological findings of RDD also showed features resembling IgG4-RD: IgG4-positive plasma cell enrichments were observed in all RDD mimic IgG4-RD patients, and the proportion of IgG4/IgG in tissues was 10–40% in 4 patients and more than 40% in 2 patients. However, none of the RDD mimic IgG4-RD patients or RDD patients displayed obliterative phlebitis or storiform fibrosis. Most of the RDD mimic IgG4-RD patients were treated with glucocorticoids combined with immunosuppressants, and a good prognosis was obtained following treatment.

**Conclusions:**

RDD has clinical manifestations that mimic IgG4-RD. However, detailed differences in laboratory parameters and pathological characteristics are present between these two diseases. Our study underlines the necessity to rule out RDD while diagnosing IgG4-RD using pathological findings as the identification criteria and provides advice for both differentiating these two diseases and clinical treatment of RDD mimic IgG4-RD.

## Background

IgG4-related diseases (IgG4-RDs) are a series of diseases characterized by specific clinical, serological and pathological features [1] displaying tumor-like enlargement of involved organs, elevated serum IgG4 levels, lymphoplasmacytic infiltration enriched in IgG4-positive plasma cells, obliterative phlebitis and storiform fibrosis. Currently, tissue biopsy remains the cornerstone for the diagnosis of IgG4-RDs [2]. As people dig deeper into these diseases, similar pathological features are discovered between IgG4-RDs and other diseases, such as Castleman disease (multicentric or localized), ANCA-associated vasculitis and RDD. According to the international consensus on IgG4-RDs (guidance statement on the management and treatment of IgG4-RDs) updated in July 2015, it is underlined that conditions mimicking IgG4-RDs need to be excluded when diagnosing IgG4-RDs [3]. RDD, also known as sinus histiocytosis with massive lymphadenopathy (SHML), is a benign lymphoproliferative disease in which pathological changes are mostly discovered in lymph nodes. In rare cases, RDD manifests at extranodal sites and mimics IgG4-RDs clinically, serologically and histopathologically by inducing enlargement and sclerosis of organs, elevation of serum IgG4 concentration, and dense infiltration of IgG4-positive plasma cells, respectively. Consequently, it is possible to misdiagnose RDD as IgG4-RD in some cases. The previous perspective that RDD and IgG4-RDs can coexist in the same patient should be reconsidered since data indicate that pathology is a dependable method to distinguish one from the other [4]. We summarize cases of RDD mimicking IgG4-RDs diagnosed at the Peking Union Medical Collage Hospital (PUMCH) and compare the pathological features of RDD and IgG4-RDs to provide further knowledge and understanding of these diseases for clinicians.

## Results

### Demographics

There were 7 patients with RDD mimic IgG4-RD, all of whom were male, with a median age of 56 (29–63) years (Tables 1, 2) and a median course of disease of 12 (2–180) months. Additionally, 10 patients were diagnosed with RDD by histopathology and immunostaining, among whom, 7 were males and 3 were females, with a median age of 52.5 (28–81) years (Table 2). Thirty-four IgG4-RD patients were selected as controls, of whom, 23 were males and 11 were females, with a median age of 54 (21–75) years (Table 2).

**Table 1:** Clinical, serology and pathology features of 7 RDD mimic IgG4-RD patients

Sequence number	Sex	Age	Organ involvement/symptom	Serology				Pathology		Treatment	Prognosis
				IgG1 (mg/L)	IgG2 (mg/L)	IgG3 (mg/L)	IgG4 (mg/L)	IgG4 (cells/hpf)	IgG4/IgG (%)		
1	M	56	Spinal pachymeningitis/numbness, walking unstable	5380	5960	420	4385	> 50	10–30	Glucocorticoids + cyclophosphamide	Improved
2	M	63	Liver mass, rash, lacrimal gland swollen	6450	7570	160	6410	10–20	10	Glucocorticoids + cyclophosphamide	Improved
3	M	61	Intraspinal mass/skin numbness	5380	5960	420	5800	> 50	40	Glucocorticoids + cyclophosphamide/azathioprine	Improved
4	M	50	Pachymeningitis/impaired vision and hearning	8000	2030	110	292	30	20	Glucocorticoids + rituximab (intrathecal injection)	Improved
5	M	29	Pachymeningitis (epilepsia)/subcutanous mass	8200	4230	318	1360	> 50	40	Glucocorticoids + cyclophosphamide	Improved
6	M	56	Pancreas and duodenum masses/lymph node enlargement	12,600	12,800	763	54,100	> 100	> 40	Glucocorticoids	Improved
7	M	37	Sellar region and orbital cavity mass/impaired vision, sinusitis	8310	5880	582	3510	> 100	> 40	None	Remain the same

M, male

**Table 2:** Clinical features of patients with RDD mimic IgG4-RD, RDD or IgG4-RD

	RDD mimic IgG4-RD (%)	RDD (%)	RDD total (%)	IgG4-RD (%)	*p *value^a^
Age (years)	56(29–63)	52.5(28–81)	52.5(28–81)	54(21–75)	–
Sex (M/F)	7/0	7/3	14/3	21/13	–
Site of involvement					
Lacrimal and salivary glands (LSG)	2/7(28.6)	1/10(10.0)	3/17(17.6)	21/34(61.8)	0.0065
Lymph node	1/7(14.3)	5/10(50.0)	6/17(35.3)	20/34(58.8)	0.1441
Pancreas	1/7(14.3)	0/10(0.0)	1/17(5.9)	8/34(23.5)	0.2412
Liver	1/7(14.3)	0/10(0.0)	1/17(5.9)	1/34(2.9)	> 0.9999
Endocranium, dura mater, sellar region	5/7(71.4)	1/10(10.0)	6/17(35.5)	1/34(2.9)	0.0038
Retroperitoneum	0/7(0.0)	0/10(0.0)	0/17(0.0)	4/34(11.8)	0.2876
Skin	1/7(14.3)	1/10(10.0)	2/17(11.8)	1/34(2.9)	0.5420

M, male; F, female

^a^Mann–Whitney test between RDD total and IgG4-RD. RDD total group includes all the patients in RDD mimic IgG4-RD group and RDD group

### Clinical features

Among the 7 patients with RDD mimic IgG4-RD, predominant disease involvement was found in the nervous system, affecting 5 out of 7 patients. Nevertheless, central nervous system involvement rarely occurred in the IgG4-RD group (1/34, 2.9%) (35.5% vs 2.9%, *p* = 0.0038, Mann–Whitney test between the total RDD group and the IgG4-RD group) (Table 2). An apparent difference was found between the total RDD group and the IgG4-RD group regarding the involvement of lacrimal and salivary glands (LSGs) (17.6% vs 61.8%, *p* = 0.0065, Mann–Whitney test), supporting previous results that LSGs are frequently affected in IgG4-RD. A similar pattern was also observed for pancreas involvement, which is an organ commonly affected in IgG4-RDs (8/34, 23.5%) but rarely affected in RDD (1/17, 5.9%). A typical clinical characteristic of RDD is the enlargement of lymph nodes. However, there was no remarkable difference in lymph node involvement between the total RDD (6/17, 35.3%) and IgG4-RD (20/34, 58.8%) groups (*p* = 0.1441, Mann–Whitney test). Frequencies of the involvement of other sites, including the liver, retroperitoneum and skin, were also analyzed in each group, but no statistically significant difference was found.

### Laboratory examination

Laboratory examinations were performed using patient serum for the RDD mimic IgG4-RD group and IgG4-RD group at the onset of disease.

**Fig. 1:**
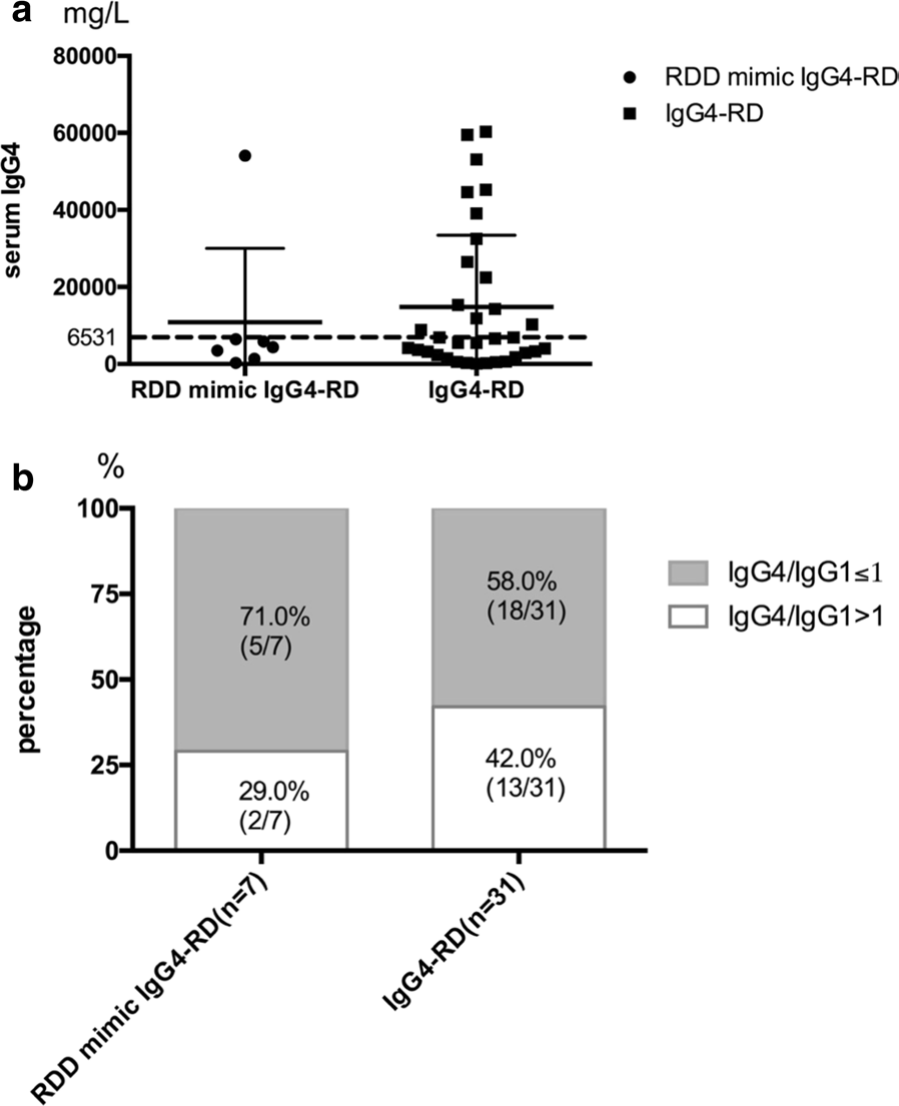
Serum IgG4 of patients with RDD mimic IgG4-RD and IgG4-RD. **a** Serum IgG4 levels in RDD mimic IgG4-RD patients and IgG4-RD patients. **b** Percentages of different ratios of serum IgG4 to IgG1 in RDD mimic IgG4-RD patients and IgG4-RD patients. Grey: percentages of patients with a ratio of serum IgG4 to IgG1 no more than 1. White: percentages of patients with a ratio of serum IgG4 to IgG1 more than 1.

Elevated serum IgG4 level (> 1350 mg/L) was found in 6 patients in the RDD mimic IgG4-RD group, ranging from 1360 to 54,100 mg/L (Table 1), and the mean serum IgG4 level in this group was 10,836.7 ± 17,780.3 mg/L (Fig. 1a). However, serum IgG4 level was still lower than that in the IgG4-RD group, in which 28 patients had elevated serum IgG4 levels (28/34, 82.4%), and the mean serum IgG4 was 15,926.3 ± 18,490.9 mg/L (Fig. 1a).

If we set the cut-off value for serum IgG4 level as > 6531 mg/L (approximately five-fold of the upper limit of normal, calculated using ROC curves) to distinguish RDD mimic IgG4-RD from IgG4-RD, the specificity is 85.71%, while the sensitivity is only 50.00% (Fig. 1a).

We also compared the proportion of serum IgG4 to IgG1 to characterize the elevation in serum IgG4 alone. We defined elevation in serum IgG4 alone as IgG4/IgG1 > 1. In our analysis, elevated serum IgG4 was discovered in approximately half of the IgG4-RD patients (42.0%, 13/31), while only 29% (2/7) of the RDD mimic IgG4-RD patients showed this serological phenotype (Fig. 1b).

**Fig. 2:**
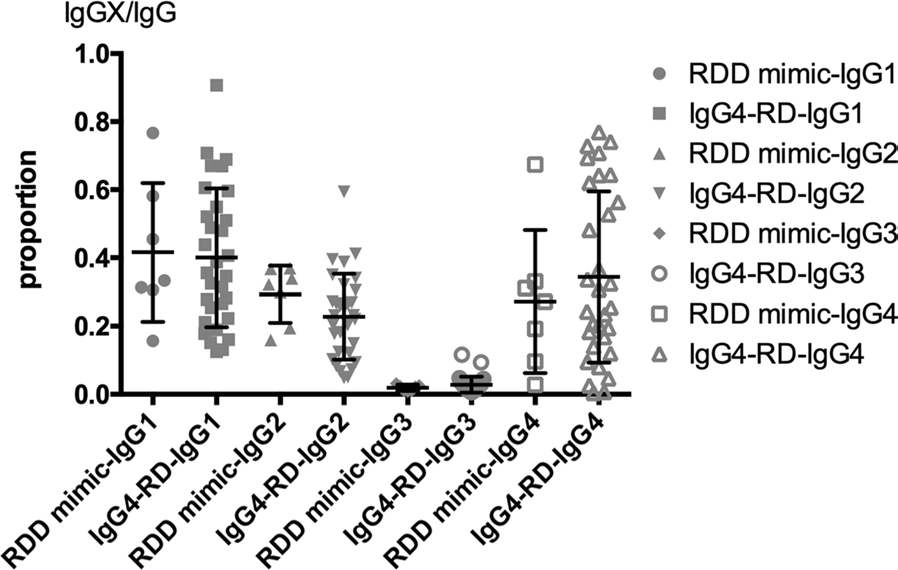
Proportion of different isotypes of IgG to total IgG in serum in RDD mimic IgG4-RD group and IgG4-RD group. IgGX refers to IgG1/2/3/4. RDD mimic-IgGX: serum IgGX to IgG in RDD mimic IgG4-RD group. IgG4-RD-IgGX: serum IgGX to IgG in IgG4-RD group.

By analyzing other IgG subclasses, we found higher IgG2/IgG levels in the RDD mimic IgG4-RD group (29.3 ± 7.7%) than in the IgG4-RD group (22.7 ± 12.3%) (*p* = 0.1343, Mann–Whitney test) (Fig. 2). A cut-off value of 28.6% for IgG2/IgG was obtained using the ROC curve to distinguish RDD mimic IgG4-RD from IgG4-RD, with a sensitivity of 74.19% and a specificity of 71.43%. This suggests a difference in another IgG subclass, IgG2, between these two diseases and the possible reason for this is mentioned in the discussion.

General laboratory parameters, including complete blood count, urinalysis results, liver and renal function indicators, erythrocyte sedimentation rate (ESR), blood eosinophil count and high-sensitivity C-reactive protein (hs-CRP), were determined in the RDD mimic IgG4-RD and IgG4-RD groups, and no significant differences were found between them. A group of antibodies, such as ANA, anti-ds-DNA Ab, anti-ENA Ab, ACA, anti-β2 glycoprotein Ab, LAC, ANCA, were also tested, and all displayed negative results.

### Histopathological characteristics

Previous immunohistochemical studies in RDD patients revealed strong reactivity in histiocytes using antibodies against S100 protein, and markers of macrophage lineage (CD14, CD68, KiM1P) showed mid to high expression, while CD1a antigen staining was negative. Another pathological feature of RDD, emperipolesis, which is the engulfment of mature lymphocytes, monocytes, plasma cells, few red blood cells and sporadic neutrophils by histiocytes with abundant cytoplasm, is also frequently observed. Previous reports on RDD have demonstrated lymphocytic aggregation with abundant plasma cells and sclerosis in affected tissues in most RDD cases, both nodal and extranodal. Furthermore, IgG4 expression from plasma cells in extranodal RDD was reported in several cases [5–9].

In our study, pathological analysis was performed on all patients using affected tissues, including the dura mater, endocranium, liver and skin. Lymphoplasmacytic infiltration enriched in IgG4-positive plasma cells (> 10 cells/hpf) was a feature found in all samples from RDD mimic IgG4-RD patients, and 3 patients (3/7, 42.9%) showed more than 50 IgG4 + plasma cells/hpf, and another 2 patients (2/7, 28.6%) showed more than 100 IgG4 + plasma cells/hpf (Table 3).

**Table 3:** Histopathological features of patients with RDD mimic IgG4-RD, RDD or IgG4-RD

	RDD mimic IgG4-RD (%)	RDD (%)	RDD total (%)	IgG4-RD (%)	*p* value^a^
IgG4-Positive Plasma Cells/hpf					
> 100	2/7 (28.6)	0/10 (0.0)	2/17 (11.8)	8/34 (23.5)	0.4632
> 50	3/7 (42.9)	6/10 (60.0)	9/17 (52.9)	19/34 (55.9)	> 0.9999
10–50(including 50)	2/7 (28.6)	2/10 (20.0)	4/17 (23.5)	7/34 (20.6)	> 0.9999
≤ 10	0/7 (0.0)	2/10 (20.0)	2/17 (11.8)	0/34 (0.0)	0.1067
IgG4/IgG					
> 40	2/7 (28.6)	5/10 (50.0)	7/17 (41.2)	28/34 (82.4)	0.0045
10–40(include 40)	4/7 (57.1)	4/10 (40.0)	8/17 (47.1)	6/34 (17.6)	0.0446
≤ 10	1/7 (14.3)	1/10 (10.0)	2/17 (11.8)	0/34 (0.0)	0.1067
Specific pathology features					
Storiform fibrosis	0/7 (0.0)	0/10 (0.0)	0/17 (0.0)	17/34 (50.0)	0.0003
Obliterative phlebitis	0/7 (0.0)	0/10 (0.0)	0/17 (0.0)	2/34 (5.9)	0.5467
Eosinophilic infiltrate	0/7 (0.0)	0/10 (0.0)	0/17 (0.0)	6/34 (17.6)	0.1612

^a^Mann–Whitney test between RDD total and IgG4-RD. RDD total group includes all the patients in RDD mimic IgG4-RD group and RDD group

**Fig. 3:**
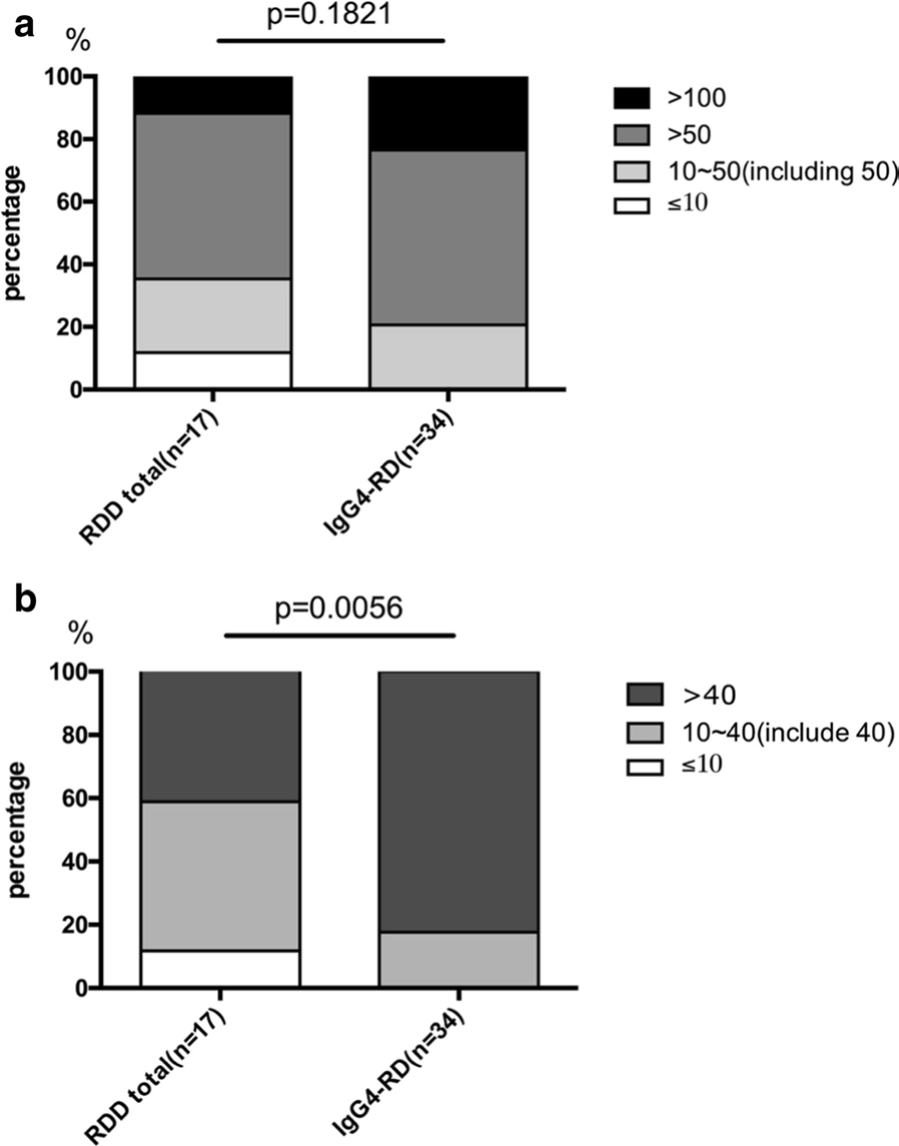
Histopathology results of IgG4-RD, RDD mimic IgG4-RD and RDD patients. RDD total includes all the patients in RDD group and RDD mimic IgG4-RD group. Chi square test was performed between RDD total group and IgG4-RD group. **a** In RDD total group and IgG4-RD group, patients that have a histopathology result with different numbers of IgG4+ plasma cells/hpf are represented with colors of different grey levels. 4 colors from dark to light: percentage of patients with a histopathology result of > 100 IgG4+ plasma cells/hpf; > 50 and ≤ 100 IgG4+ plasma cells/hpf; > 10 and ≤ 50 IgG4+ plasma cells/hpf; ≤ 10 IgG4+ plasma cells/hpf. **b** In RDD total group and IgG4-RD group, patients with different ratios of IgG4/IgG in tissue in histopathology analysis are represented with colors of different grey levels. 3 colors from dark to light: percentage of patients with a ratio of IgG4/IgG > 40; with a ratio of IgG4/IgG > 10 and ≤ 40; with a ratio of IgG4/IgG ≤ 10.

We compared IgG4 + plasma cell enrichment in the total RDD group to that in the IgG4-RD group but found no statistically significant difference between these two groups (*p* = 0.1821, chi-square test), although there was a trend toward more IgG4 + plasma cell enrichment in the IgG4-RD group, with a higher percentage of patients (27/34, 79.4%) in the IgG4-RD group with more than 50 IgG4 + plasma cells/hpf (Fig. 3a). For IgG4/IgG, a statistically significant difference was found between the total RDD group and the IgG4-RD group (*p* = 0.0056, chi-square test) (Fig. 3b), affirming a tendency for more IgG4 + plasma cells in the IgG4-RD group. Two patients (2/17, 11.8%) in the total RDD group had a proportion lower than 10%, while this feature was not observed in any patient in the IgG4-RD group. Moreover, 82.4% (28/34) of patients in the IgG4-RD group had a proportion of more than 40%, while this feature was only observed in 41.2% (7/17) of patients in the total RDD group. Based on these significantly different immunostaining results in the two groups, RDD patients displayed IgG4 + plasma cell enrichment similar to but still slightly lower than IgG4-RD patients. We also analyzed specific pathological features of IgG4-RD, including storiform fibrosis, obliterative phlebitis and eosinophilic infiltration, in all patients. Most IgG4-RD patients had at least one of these features as predicted (Table 3). On the other hand, as expected, none of the RDD patients displayed these pathological features. These data suggest that specific pathological features of IgG4-RD are reliable criteria for diagnosing IgG4-RD while ruling out RDD.

### Treatment and prognosis

Among the 7 patients with RDD mimic IgG4-RD, 6 were started on treatment with glucocorticoids, while one patient did not receive any treatment due to the patient’s decision. Five of these 6 patients also received an immunosuppressor therapy, including cyclophosphamide, azathioprine and rituximab (Table 1). The condition of all of the patients who received treatments improved during follow-up. However, some organ damage, such as vision and hearing loss, was irreversible.

## Discussion

In our study, elevated serum IgG4 levels and infiltration of IgG4 + cells in tissues were observed in RDD patients with either nodal or extranodal involvement, mimicking IgG4-RDs clinically, serologically and pathologically. This underlines the necessity to distinguish RDD from IgG4-RDs in our clinical work. Our study analyzed a group of patients with RDD mimic IgG4-RD and characterized the clinical, laboratory and histopathological features of RDD and IgG4-RD. Extranodal involvement was found in both diseases. However, RDD mimic IgG4-RD was likely to involve the nervous system but rarely to involve LSGs, which are commonly affected in IgG4-RD. RDD mimic IgG4-RD patients had an elevated serum IgG4 level, but the level was still lower than that in IgG4-RD patients in general. Pathologically, there were features that remained exclusively to either RDD or IgG4-RD, which could be regarded as the gold standard for distinguishing one disease from the other. Moreover, although we discovered enrichment of IgG4 + plasma cells in affected tissues in RDD, a small gap remained between RDD and IgG4-RDs with regard to the proportion of IgG4 + plasma cells.

Rosai–Dorfman disease, or sinus histiocytosis with massive lymphadenopathy, is a rare, benign histiocytic proliferative disease. It occurs predominantly in children and youth, manifesting as chronic inflammation and painless lymph node masses, and extranodal involvement is discovered in 25% to 40% of patients [10]. Characteristic pathological changes associated with this disease are phagocytosis of lymphocytes (emperipolesis) and the proliferation of S-100-positive histiocytes.

IgG4-RD is an immune-mediated condition that is likely to form tumorous lesions in multiple organs and is characterized by elevated serum IgG4 level and IgG4-positive plasma cell infiltration in affected tissues. However, these two features, both serological and pathological, are observed in many other diseases, suggesting that these diseases may mimic IgG4-RD.

Consistent with previous studies, our study showed that RDD could mimic IgG4-RD. We thus analyzed three groups of patients clinically, serologically and pathologically.

Clinically, for RDD with extranodal involvement, masses and polyploid protrusions are the most common presentation. It is difficult to distinguish these clinical manifestations from those of IgG4-RD, which usually presents as organ enlargement and sclerosis. The most commonly affected extranodal site in RDD is the skin. Other organs that may be affected include the central nervous system, soft tissues, the upper respiratory tract, the nasopharynx, breasts, testes, kidneys, thyroid glands and the appendix [11–14]. For IgG4-RD, the organs most often involved are LSGs, the pancreas, the bile duct, the retroperitoneum, thyroid glands, kidneys and lungs. We obtained the same results as previous studies showing that extranodal involvement often occurs in the nervous system in RDD, while LSGs are the sites mostly involved in IgG4-RD. However, contrary to previous studies that indicate that most RDD patients have lymph node involvement, our study showed no statistically significant difference in lymph node involvement between RDD and IgG4-RD. This may be due to the way the patients in the total RDD group were diagnosed. In addition to the 7 patients with RDD mimic IgG4-RD, all of the patients had extranodal involvement, and patients in the RDD group were mostly recruited from surgery departments and diagnosed based on histopathology postsurgery; thus, these patients were likely to have extranodal involvement, which resulted in a lower percentage of RDD patients with lymph node involvement. Overall, we may roughly distinguish these two diseases based on their distinct clinical features. However, the RDD patients in our study also displayed lacrimal gland swelling, which may misguide the diagnosis of IgG4-RD. Therefore, clinical manifestations can only provide indications but not solid evidence for diagnosis.

With regards to laboratory parameters, elevated serum IgG4 concentration is recognized as the dominant serological characteristic of IgG4-RD. However, reliance upon this feature for establishing the diagnosis of IgG4-RD is usually highly problematic because of the poor specificity (60%) when the cut-off value of serum IgG4 concentration is set at > 1350 mg/L. Many studies have reported that serum IgG4 levels could be increased in various diseases, including connective tissue disease, chronic infection, lymphoma, and Castleman disease, making this parameter unreliable as the only criterion for the diagnosis of IgG4-RD. Our results support this notion, showing that 6 out of 7 RDD mimic IgG4-RD patients had elevated serum IgG4 levels. However, we also found that patients with RDD mimic IgG4-RD had a lower level of serum IgG4 levels than IgG4-RD patients. Furthermore, we tried to set a new cut-off value of serum IgG4 levels to distinguish these two diseases. Research indicates that the cut-off value should be adjusted in some situations to improve the diagnosis of IgG4-RDs [15]. Since the present cut-off value may perform even more poorly when used to distinguish IgG4-RD from diseases mimicking it, in our study, we discovered the cut-off value of serum IgG4 level > 6531 mg/L using a ROC curve, performed for data in Fig. 2, which had high specificity (85.71%) but low sensitivity (50%). This value could be important for diagnosing patients with diseases mimicking IgG4-RD. It is found in clinical practice that the elevation in serum IgG4 concentration is more significant when other IgG isotypes remain normal, but this feature may not be so meaningful if the concentrations of all IgG isotypes are elevated. Along those lines, elevation in IgG4 alone was found in our study in IgG4-RD patients. Another interesting result was that a higher serum IgG2/IgG level was found in RDD mimic IgG4-RD patients than in IgG4-RD patients. Serum IgG2 is usually produced in response to bacterial capsular polysaccharide antigens [16–18]. Elevated serum IgG2 levels were observed in some IgG4-RD cases [19], but it has never been studied in RDD. It has been reported that the class switching of the IgG subclass proceeds through an ordered pattern from IgG3 to IgG1 to IgG2 to IgG4 [20]. Therefore, one possibility for the elevated IgG2/IgG proportion is that IgG2 in produced en route to IgG4, and the speed of class switching may be different between RDD and IgG4-RD. Laboratory test results in our study suggested that as serum IgG4 level becomes specifically higher, there is a higher possibility to diagnose IgG4-RD, but this serum feature is not sufficient to rule out RDD.

Recently, the presence of IgG4 + plasma cells has been pathologically discovered in a considerable number of RDD cases. Research performed by Zhang et al. [5] demonstrated that of 26 RDD cases, 73.1% exhibited more than 10 IgG4 + plasma cells/hpf, and 46.2% displayed more than 30 IgG4 + cells/hpf. Meanwhile, in tissues, the IgG4/IgG proportion in some RDD cases could be as high as in IgG4-RDs [6, 21]. Because of the similar histopathological features, these 2 diseases are speculated to fall within the same [7] or overlapping [22] disease spectrum. However, others consider that RDD is not a kind of IgG4-RD because in affected tissues, IgG4 + plasma cells, IgG4/IgG proportion and foxp3 + Tregs are still lower than those in classic IgG4-RD but similar to those in reactive hyperplastic lymph nodes [10]. The results in our study display apparent enrichment of IgG4 + plasma cells and elevated IgG4/IgG ratio in RDD patients, which mimic the pathological characteristics of IgG4-RD. Although we found that the infiltration of IgG4 + plasma cells in RDD was not as pronounced as that in IgG4-RD, no convincing standard for diagnosis could highlight this feature.

Nonetheless, IgG4-RDs and RDD display diverse and specific pathological features. Pathological features, including storiform fibrosis, obliterative phlebitis and eosinophilic infiltration, were found specifically in IgG4-RD patients but absent in RDD patients in our analysis, consistent with findings in previous studies [10]. These characteristics are of great importance because, as emphasized in the consensus statement on the pathology of IgG4-RDs [4], these are key for an unequivocal diagnosis of IgG4-RDs, even if IgG4 + plasma cell infiltration is confirmed. On the other hand, pathological characteristics exclusive to RDD were also confirmed in our study, including S-100( +), CD38( +), CD68( +) and emperipolesis in histopathological analysis in all samples from the RDD mimic IgG4-RD group but not in the IgG4-RD group. In recent years, studies of RDD using next-generation sequencing found neoplastic conditions, point mutations occur in 33–50% of RDD cases, affecting KRAS, NRAS, SMAD4, ARAF, or MAP2K1, which is not found in IgG4-RD [23–25]. This gives a possible way for differentiating RDD mimic IgG4-RD using next-generation sequencing in some cases. These findings all support the notion that these 2 diseases fall along distinct spectra, and a clear separation of these diseases should be made during diagnosis.

Treatment is different for RDD and IgG4-RD. As most IgG4-RDs show a good response to corticosteroids, the treatment for RDD varies according to the severity of the disease. Some of the RDD cases are self-limited and no treatment is required, and some can be controlled using corticosteroids, while others are lymphoma-like and need chemotherapy. As for RDD mimic IgG4-RD, in these cases, response to corticosteroids is not as good as it is in IgG4-RD, and most of them need treatment of high intensity, such as combining with immunosuppressive drug or using chemotherapy. Therefore, diagnosing RDD from IgG4-RD correctly, especially for those mimicking IgG4-RD clinically and pathologically, can lead to standard treatment with better prognosis.

There are some limitations in our study regarding time of follow-up and number of patients. Firstly, the number of patients with RDD mimic IgG4-RD is limited in our study. Because both RDD and IgG4-RD are rare diseases, very few numbers of patients could fulfill our requirements and be enrolled in our research, leaving the problem of not enough samples to be analyzed. A study with more patients is warmly welcomed to give a better cut-off value of serum IgG4 to distinguish these two diseases. Secondly, follow-up time for prognosis is not long enough. In our study, the follow-up time period for each patient post diagnosis is no longer than 1 year, which is adequate to notice the improvement after treatment but not long enough to discover a relapse of the disease. Further study with a longer period of follow-up should be conducted to estimate long-term prognosis.

## Conclusions

In conclusion, some RDD cases mimic IgG4-RDs in terms of clinical manifestations, laboratory parameters and pathological characteristics. However, since both of these diseases have specific pathological features, we speculate that these two diseases fall along distinct spectra, emphasizing the necessity to rule out RDD when suggesting a definitive diagnosis of IgG4-RD. Special attention should be paid to the level and proportion of serum IgG4 and the number and proportion of IgG4 + plasma cells on histopathology, and above all, the respective pathological characteristics for these two diseases are the key step for diagnosing. Because RDD mimic IgG4-RD tends to involve sites that may result in severe symptoms, aggressive therapy is recommended for these cases.

## Materials and methods

### Patient enrollment

We prospectively enrolled 7 patients who were suspected to have IgG4-RD but were finally diagnosed with RDD (the RDD mimic IgG4-RD group) in the Rheumatology Department, PUMCH from January 2012 to March 2018. Another 10 patients diagnosed with RDD in other departments (including the Otolaryngology Department, Stomatology Department, Department of Plastic Surgery, Orthopedics Department and Department of Cardiac Surgery) during the same period were retrospectively enrolled in our study (the RDD group). Serum IgG4 level was not estimated at the onset of disease for these 10 patients, but immunohistochemical staining of IgG4 and IgG was performed on samples from affected tissues. We classified the 7 patients in the RDD mimic IgG4-RD group and the 10 patients in the RDD group together as the total RDD group, which contained 17 patients. Thirty-four IgG4-RD patients who were matched to the 17 RDD patients by age and sex were randomly selected at a ratio of 2:1 from our IgG4-RD cohort as controls.

### Methods for investigation

We analyzed general conditions, clinical symptoms and signs, laboratory parameters, histopathological features, treatment and prognosis of the patients and compared the differences among the groups.

### Statistical analysis

Statistical analysis was performed using GraphPad Prism 7 (GraphPad Software v7.1). Continuous normally distributed data were displayed as the mean ± standard deviation and analyzed by parametric tests. Continuous nonnormally distributed data were displayed as the mean ± standard deviation and analyzed by nonparametric tests. Discrete nonnormally distributed data were presented as medians (minima–maxima) and analyzed by nonparametric tests.

## Data Availability

The relevant data used to support the findings of the study are included within the article.

## Abbreviations

RDD: Rosai–Dorfman disease
IgG4-RD: IgG4-related disease
RDD mimic IgG4-RD: Rosai–Dorfman disease mimicking IgG4-related disease
LSG: Lacrimal and salivary gland
